# The Role of PET-CT-Guided Metabolic Biopsies in Improving Yield of Inconclusive Anatomical Biopsies: A Review of 5 Years in a Teaching Hospital

**DOI:** 10.3390/diagnostics13132221

**Published:** 2023-06-29

**Authors:** Dharmender Malik, Vineet Pant, Ishita Sen, Parul Thakral, Subha Shankar Das, Virupakshappa CB

**Affiliations:** Department of Nuclear Medicine, Fortis Memorial Research Institute, Gurgaon 122002, India; dr.vineet.pant@gmail.com (V.P.);

**Keywords:** 18F-FDG PET-CT, metabolic biopsies, oncology, pneumothorax, percutaneous biopsies, CT-guided biopsies

## Abstract

Tumour sampling is indispensable to diagnostic and therapeutic decision making. Thus, 18F-FDG PET/CT has the potential to accurately discriminate between viable and non-viable tissues due to its ability to characterise the metabolism of visible tissues. This study’s objective was to evaluate the incremental utility of 18F-FDG PET-CT-guided metabolic biopsy in individuals with suspected lesions and a previous negative anatomical biopsy. This study included a total of 190 consecutive patients with probable malignancy and who had experienced a previous unsuccessful anatomical biopsy who underwent PET-CT-guided metabolic biopsy. We retrospectively analysed the patients’ medical records and imaging investigations to assess demographics, complications, pathologies, and final clinical diagnoses. Using multivariate logistic regression, correlation between several confounding factors that lead to post-procedural problems was evaluated. Adequate material was obtained in all patients, and 162 (85%) were found to be positive for malignancy with a diagnostic yield of 96.9%. In 25 (13.1%) patients, post-procedural complications were reported, with pneumothorax being the most prevalent issue. In evaluating oncological patients, metabolic biopsy provides a safer alternative therapy with a high diagnostic yield and comparable complications. PET-CT, being an essential component of cancer staging, may serve as a one-stop shop for the management of these patients’ conditions.

## 1. Introduction

18F-FDG PET-CT is currently regarded as an indispensable part of the evaluation of oncological patients due to its capacity to better characterise malignancy and added usefulness in correctly staging patients whose malignancy is later confirmed [1]. In oncology, tissue sampling is essential for making correct diagnostic and therapy decisions. Since its introduction, percutaneous computerised tomography (CT)-guided needle biopsies have received widespread acceptance in a variety of therapeutic contexts [2,3]. CT-guided biopsies are generally a reliable method for obtaining tissue samples; however, in a significant number of instances, the tissue obtained may not be representative of the underlying disease, which may be due to CT’s inability to differentiate between viable and non-viable necrotic tissue, resulting in false-negative results [4,5]. In contrast, the 18F-FDG PET scan can investigate the metabolic features of masses. Consequently, if PET data could be incorporated into a CT-guided biopsy method, the yields of biopsies from such lesions might possibly be increased. This goal can be accomplished either by co-registering previously acquired PET data with intraprocedural CT data, or running an intraprocedural single-bed PET CT acquisition of the region of interest and utilising this dataset to guide the biopsy [6,7]. While the first method is technically applicable to the majority of existing co-registration software, it is susceptible to registration errors due to patient placement, movement, and respiration issues. As a result, precise targeting is difficult, treatments may be extended, and patient and medical staff radiation exposure may be increased. Although technically hard, performing a single bed intra-procedural PET CT capture and using this dataset to guide the biopsy may reduce these errors, while also increasing the diagnostic yield of a CT-guided biopsy [8,9]. We developed a protocol for multimodal image-guided interventions that utilises an integrated PET-CT machine; here, we report our experience in a retrospective analysis of the cases in which this protocol was used to perform 18F-FDG PET-CT-guided percutaneous metabolic biopsies of suspected oncological patients who had registered previous negative results during anatomical biopsies.

## 2. Material and Procedures

### 2.1. Inclusion Criteria

This study included patients with high clinical suspicion of malignancy and had at least one failed biopsy guided via conventional radiological imaging. The reasons for the failure were analysed by a multidisciplinary team (MDT), which included an interventional radiologist, a nuclear medicine physician, and a treating oncologist. A PET-guided biopsy was only ordered for those cases in which the MDT felt that repeated conventional imaging-guided biopsy, even after eyeballing the PET CT images was available, was likely to fail to produce representative samples. Based on this stringent inclusion criteria, 194 consecutive patients underwent percutaneous metabolic biopsy guided via PET-CT between 2018 and 2022. We retrospectively analysed the patients’ medical records and imaging data in order to ascertain the patient’s demographics, procedural characteristics, complications, pathologies, and final clinical diagnoses. Patients with one or multiple suspected lesions that demonstrated FDG uptake through a previously acquired PET-CT and whose prior anatomical (CT/USG-guided) biopsy was unable to give clear results due to tissue necrosis or inadequate sampling met the inclusion criteria. Four cases were eliminated due to a lack of complete information. The final study population consisted of 190 patients, with a mean age of 52.5 years (range: 8 to 92 years), that contained 113 men and 77 women. This study was authorised by an Institutional Review Board and conducted in conformity with applicable ethical standards. PET CT-guided procedures were performed by nuclear medicine specialists who were trained to perform image-guided biopsies. Each physician was required to have completed at least 30 CT-guided percutaneous biopsies in order to participate in this study.

### 2.2. PET CT-Guided Technique for Biopsy

Written informed consent was obtained from patients before performing metabolic biopsies. Prior to the biopsy, the patient’s PT, INR, and platelet counts were evaluated, and the biopsy was only performed if the INR was less than 1.5 and platelet counts were adequate. The individuals were injected with 74 to 148 MBq of 18F-FDG, and the biopsy was performed 90 to 120 min after isotope injection. In certain situations where a diagnostic whole-body PET-CT scan was performed on the same day as the biopsy, the PET-CT-guided metabolic biopsy was performed as a second procedure within 4 h of radioactive administration. A PET-CT scan with a single bed was performed on the region of interest. The site of needle entry, the access path, and the direction of approach for biopsy were determined based on the presentation of the metabolically active lesion of interest in this PET-CT dataset, which gave the most direct route for biopsy. Patients were positioned prone, supine, or in a posture of lateral decubitus closest to the body surface.

In each instance, a 20-gauge coaxial needle with an 18-gauge outer trochar was utilised. Depending on the calculated distance of the lesion from the place of needle entrance, the needles varied in length from 9 to 15 cm. For regional anaesthetic, a 2% lidocaine solution was employed. In the biopsy’s location, the needle was introduced using a step-by-step procedure tailored to the specific anatomical landmarks seen on the CT dataset. Before sampling, the correct needle position in the centre of the FDG avid lesion was ensured by executing a 60-s single-bed PET-CT acquisition. The metabolically active region of the lesion was precisely targeted, and representative samples were collected. All patients underwent FNA biopsies, tissue core biopsies, or a combination of the two methods. Whenever a FNA biopsy was conducted, a cytopathologist was present to confirm the sample’s appropriateness. After each biopsy, the sample was sent to the on-site cytopathologist for analysis of its yield. Half of the aspirated FNA samples were stained with Dif-Quick for on-site evaluation, while the other half were fixed in 95% ethyl alcohol for laboratory evaluation. Rarely were repeat FNAC tests performed, as a core biopsy was conducted on each occasion. After being placed in formalin, the tissue-core biopsy specimens were sent out to be embedded in paraffin, and tissue sections were then taken from them. When an infectious lesion was suspected, the smears were also stained to identify micro-organisms, such as acid-fast bacilli or fungi. Aspirates were also diluted with normal saline and sent for culture analysis.

### 2.3. Post-Biopsy Remarks

After the biopsy needle was removed, a CT scan of the same site was performed to detect any complications. The patients were then instructed to lie with the biopsy side facing downwards for at least 2 h in the observation bay of the nuclear medicine department, before being discharged.

In patients with lung lesions, a pneumothorax was diagnosed on the post-biopsy check scan if the pleural surface seemed to retract from the parietal pleura. After 4 h of observation, asymptomatic patients with pneumothorax who demonstrated no advancement of pneumothorax during a follow-up chest X-ray or were clinically asymptomatic were discharged. Patients who experienced moderate or severe pneumothorax were treated by inserting a chest tube.

Hemoptysis was considered a problem if it happened during or after a biopsy and there was no previous history of hemoptysis. Post-biopsy hemoptysis was classified as either non-life-threatening (i.e., not causing abnormal gas exchange/airway obstruction or hemodynamic instability) or life-threatening hemoptysis (i.e., causing abnormal gas exchange/airway obstruction and/or hemodynamic instability). A haemothorax was identified when a biopsied specimen revealed fresh fluid accumulation in the pleural area with an attenuation of 30 to 40 HU.

### 2.4. Radiation Dose to the Operator

The physicians performing the biopsy carried a pocket dosimeter to measure the total radiation dose received by the operator during the PET CT-guided procedure. A record was also made of the overall time required for the procedure, which was determined as the time between the start of the acquisition of the first CT in the biopsy position and the end of the acquisition of the check CT following the biopsy.

### 2.5. Collection of Data

The demographics of the patient, the technical details of the biopsy process, and post-biopsy problems and their management were noted. The age of the patient, lesion size, lesion depth, needle size, travelled length, and number of passes were evaluated in relation to the occurrence of post-biopsy problems. The lesion size was assessed along the maximal long-axis diameter, and lesion depth was recorded from the skin’s surface to the edge of the lesion closest to the needle path. In patients with lung lesions, adhesion of lung lesions to the pleura and the existence of emphysematous alterations in the surrounding lung parenchyma were also documented.

## 3. Analytical Statistics

A statistical software tool was employed for data analysis (SPSS version 21, IBM Corp, Armonk, NY, USA). Continuous variables were expressed as means ± standard deviations and ranges. Categorical variables were expressed as frequencies with percentages. The accuracy of biopsies for diagnosing cancer were determined.

The occurrence of post-procedural complications, such as bleeding (hematoma formation), hypotension, acute pain, infection, pneumothorax, and hemoptysis, were analysed in relation to the following procedure variables: needle-length traversed, number of passes, presence of emphysematous changes in the surrounding lung parenchyma, adhesion of the lesion to the adjacent organs, and the age of the patient; at this stage, we used a multiple logistic regression model that was constructed in a step-by-step manner. Variables that were significant in univariate analysis at *p* ≤ 0.10 were preserved for inclusion in the model in a step-wise fashion. Variables with the strongest univariate relationships were fitted into the model before other variables. Only predictor variables independently related to post-procedure complications at *p*-value ≤ 0.05 were included in the final model.

## 4. Results

**Figure 1:**
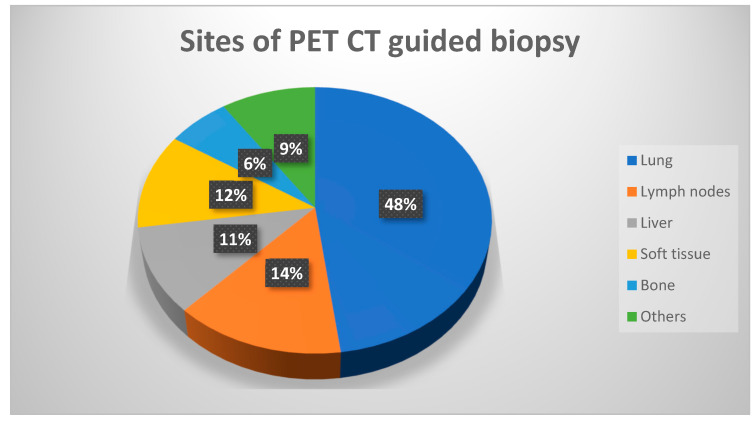
Chart demonstrating the distribution of metabolic biopsy sites.

**Figure 2:**
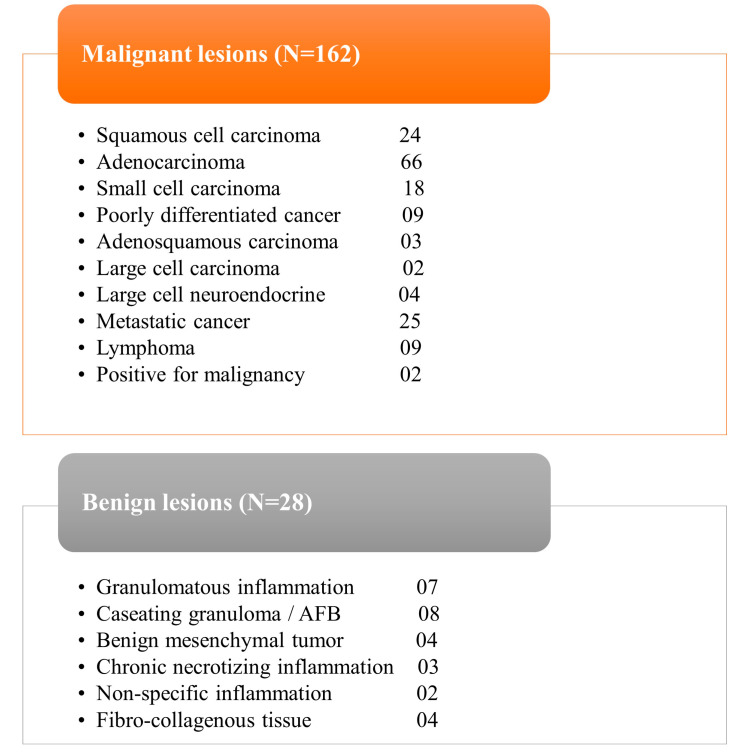
Tables demonstrating the final histopathological analysis of all patients.

The study included 190 consecutive patients who underwent percutaneous metabolic biopsies and were sent to our department after previous anatomical biopsies yielded negative results. Table 1 provides a complete summary of patients’ demographics, clinical features, and pathological findings. The average age of the patients was 52.5 (in a range of 7 to 80) years. Among the samples collected during metabolic biopsies, the lung lesions comprise the biggest cohort, followed by lymph nodes and soft tissue lesions, respectively; details of sites of biopsy are provided in Figure 1. Except for two individuals, sufficient samples were acquired from all patients. These two individuals received positive diagnoses of cancer but inadequate material for further IHC characterisation. Histopathological study reveals that 162 (85%) of the lesions were malignant and 28 (15%) were benign (Figure 2), including inflammatory cells, necrotic tissue, and infection. The patients with benign results were appropriately treated based on histology findings and monitored for at least 6 months to rule out disease development. Four biopsies (2.1% of total) yielded non-specific results and were deemed non-diagnostic. The overall diagnostic yield of metabolic biopsy in our study was 96.9%. Among the 190 metabolic biopsies performed in our study, the rate of complications (major and minor) was 13.1% (25 patients), with the greatest number occurring in patients with lung lesions (21 patients). Two individuals had life-threatening complications that contributed to their deaths (one individual had a large perihepatic hematoma and the other individual had large volume haemoptysis). The patient with severe haemoptysis had numerous coexisting morbid conditions.

Thirteen patients developed pneumothoraxes (two of which required chest drain insertion), while nine had hemoptysis (eight of which were not life-threatening and one of which was life-threatening and associated with mortality) and one evolved subcutaneous emphysema. Two patients experienced both hemoptysis and pneumothorax; however, neither of the cases were life-threatening, and they required no additional treatment. Using univariate and multivariate logistic regression analysis, a number of different independent risk factors for the prediction of post-procedural complications (PPCs) were explored (Table 2). Of these factors, the crossed needle length (>30 mm; *p* < 0.0001) and lesion-size (≤33 mm; *p* = 0.0072) were significant. There was no correlation between the post-procedural complication rate and patient age or needle size,. Multivariate analysis identified having several puncture sites, as well as the depth of the lesion, as the most significant predictors of PPCs. The incidence of hemoptysis was also strongly linked to the lung length traversed (>3 mm; *p* = 0.016) and lesion size (*p* = 0.0478). The average time recorded for the entire procedure was 26 ± 3.4 min, and the average radiation dosage exposure per procedure for the nuclear medicine practitioner was 0.014 mSv.

## 5. Discussion

By mapping hypermetabolic activity throughout the body, PET/CT is a useful imaging technique for the identification and staging of cancer. Thus, 18F-FDG PET/CT is widely utilised in the evaluation of oncological patients due to its capacity to detect malignancies prior to the emergence of morphological alterations using conventional imaging techniques that examine anatomy. The present study demonstrates the role of metabolic biopsy in providing the more accurate results in prior failed anatomical biopsies without any added risk of significant complications.

CT-guided biopsy has been widely accepted as an effective and safe procedure for diagnostic confirmation in a variety of clinical settings for several years; however, CT’s inability to distinguish between viable and necrotic tissue poses a significant challenge and results in a high number of false-negative cases [10,11]. Some cancers are not visible without using intravenous contrast material on CT scans, specifically in the hepatic malignancies (and when contrast is utilized, its effects are often temporary and not long lasting enough to allow visualisation of masses throughout the procedure). In contrast, the radiotracer (FDG/PSMA/DOTANOC etc.) becomes incorporated into the mass, remains within the lesion for a longer duration, and enables a greater window within which to perform the metabolic biopsy [12,13]. A similar phenomenon was observed, in our study, among the 20 liver lesions, 7 (35%) of which were not visualised on the CT scan, and biopsy was conducted based on highest FDG uptake. In the case of large tumours with substantial core necrotic components, PET-CT typically reveals heterogeneous radiotracer uptake. Based on metabolic information received via PET-CT, the needle can be placed into the region of a lesion with increased metabolic uptake. Similarly, the metabolic biopsy is found to be effective in lymph node chains where only a subset of lymph nodes exhibits FDG uptake and is, therefore, more likely to contain cancer cells [7,14,15]. A further advantage of PET-CT is its ability to visualise metabolic characteristics of malignant tumours prior to their morphological transformation. In prospective research undertaken by Cerci et al., 9.6% (18/188) of patients who underwent PET/CT-guided biopsies exhibited FDG-avid foci without any matching structural alterations being found on the CT scan. The authors recommended emphasising the value of FDG PET/CT data in selecting biopsy sites [16].

**Figure 3:**
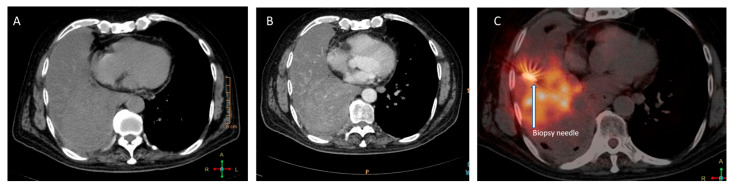
Images depicting the incremental value of metabolic biopsy in improving the diagnostic yield. A 53-year-old man, who is a known smoker, presented with near-complete collapse of the right lung with high suspicion of malignancy. The conventional CT-guided biopsy failed to provide the adequate result, as seen in figure (**A**); thus, it was impossible to distinguish between the mass and collapsed lung parenchyma, as even the contrast-enhanced CT (**B**) was unable to clearly distinguish between the two aspects. A repeat PET-CT-guided metabolic biopsy was performed from the FDG avid part of the mass (**C**), which turned out to be adenocarcinoma of lung upon histopathological examination.

The big lung masses are frequently associated with heterogeneous architecture, consolidations, atelectasis, necrosis/fibrosis, and ground glass opacities (GGO’s). These tumours may be predominantly necrotic and contain only a tiny proportion of metabolically active tumour cells. Necrosis and GGOs are substantially correlated with false-negative lesions [5,17,18]. PET/CT permits the identification of FDG-rich portions of large necrotic lesions and aids in distinguishing consolidation from live-tumour tissue (Figure 3). FDG PET/CT guides the biopsy to the most metabolically active section of the lesion, minimising sample errors that could come from biopsying areas with necrosis or fibrosis [19]. In cases of patient with multiple lesions, PET-CT scans also enable the determination of the best accessible spot for biopsy. Biopsy of the most accessible lesion can minimise sampling errors and reduce the incidence of complications associated with the procedure [9].

In the present study, lesion size and depth and needle length were found to be substantially linked to PPC risk. Multivariate analysis revealed that having multiple puncture sites and the size of the lesion were the most important risk factors for PPCs.

The biggest disadvantage of metabolic biopsy is an increased radiation exposure for the patient and the operator. Patients who experience a whole-body PET-CT receive an average radiation dose of between 8 and 30 mSv, though this dose can vary widely based on the specifics of the investigation and the number of body regions scanned. Between 54 and 81% of the total dose is typically provided by the CT portion [20,21]. During a PET/CT-guided biopsy, patients are exposed to radiation from both the initial diagnostic PET/CT scan and the subsequent CT scans collected during the biopsy. However, regardless of the biopsy, these patients would have undergone a diagnostic PET-CT scan. Therefore, the additional radiation administered to patients as a result of the biopsy is limited to the brief CT scans performed during needle placement. For patients requiring dedicated PET-CT-guided metabolic biopsy, we administered only a modest amount of 18F-FDG, with a mean dose of 102 MBq (range 74–148 MBq), to keep radiation exposure to a minimum with optimal image quality. Recently, researchers studied the utility of low- and ultra-low-dose CT protocols and demonstrated that significant reduction in radiation dose can be achieved with no detrimental influence on the diagnostic rates of the biopsy operations. Incorporating these novel CT techniques and decreasing the amount of 18F-FDG provided the bare minimum and is, thus, a partial solution to the problem of increased radiation exposure to patients [22]. In our investigation, the average radiation exposure per procedure for operating personnel was 0.014 mSv, which is far below the permitted annual limits.

The use of single-institution data and its retrospective nature are the primary limitations of our investigation. Another issue is the lack of direct comparison with the CT-guided biopsy. Performing both procedures (CT-guided and PET-CT-guided biopsies) in any particular patient is universally restrained due to ethical constraints. Also, the shortage of well-equipped PET-CT centres restricts the generalizability of this technology and challenges its potential to replace the CT-alone approach. Hence, by identifying a more selective patient population that had at least one past inconclusive biopsy result, we targeted a more promising area of research.

## 6. Conclusions

The integral value of 18F-FDG PET-CT in the workup of oncological patients, as well as its ability to detect malignancy before morphological changes manifest, suggests that PET-CT-guided interventions will likely gain traction in the near-future. PET guidance can maximise the diagnostic yield of image-guided procedures and direct needle insertion into the viable area of the lesion. These results suggest that metabolic biopsy is a practical, safe, and efficient method that produces a good diagnostic performance and, consequently, can have immediate impact on treatment decisions and patient care.

## Figures and Tables

**Table 1:** Clinical, procedure characteristic, and pathological results of patients who underwent percutaneous metabolic biopsy.

Clinical Characteristics	N = 190	N = 91
Age (years) ± SD	52.5 ± 3.1	54.1 ± 2.6
Sex		
Male	113	56
Female	77	35
Procedure characteristics		
Lesion size (cm) ± SD	3.3 ± 1.8	2.9 ± 1.9
Position		
Supine	105	59
Prone	71	32
Decubitus	14	00
Pathological results		
Adequate diagnosis	188	91
Benign	051	17
Malignant	137	74
Inadequate sample	02	00

**Table 2:** Univariate and multivariate logistic regression analyses for detection of post-procedural complications in the patients undergoing percutaneous metabolic biopsy.

	Univariate			Multivariate		
	OR	95% CI	*p*-Value	OR	95% CI	*p*-Value
Age (year)	1.003	0.97–1.04	0.98			
Sex (male)	0.915	0.86–1.45	0.71			
Lesion size (cm)	1.09	1.02–1.17	0.0072	2.19	1.56–3.24	0.07
Crossed needle length	3.5	0.90–8.51	<0.001	5.81	1.3–25.1	0.03
Patient position (supine)	0.96	0.92–1.06	0.09			
Multiple puncture sites (+)	6.13	1.48–25.81	0.013	28.4	3.18–98.05	0.009

## Data Availability

Data is unavailable publicly due to privacy concern and ethical restrictions. However it can be made available on demand.
